# Mobile Atheroma in the Left Internal Carotid Artery: A Case of Impending Doom!

**DOI:** 10.7759/cureus.51181

**Published:** 2023-12-27

**Authors:** Marc T Zughaib, Medhat Chowdhury, Andrew D Assaf, Mathhar Aldaoud, Marcel E Zughaib

**Affiliations:** 1 Department of Cardiology, Ascension Providence Hospital - Michigan State University College of Human Medicine, Southfield, USA

**Keywords:** interventional cardiology, cardiology, mobile atheroma, carotid artery disease, carotid stent

## Abstract

A 69-year-old male presented for evaluation of a carotid bruit. Carotid ultrasound demonstrated the unique finding of a large, highly mobile atheroma in the proximal left internal carotid artery. The presence of a mobile atheroma confers an even higher risk of stroke, so this presentation posed a dilemma in terms of endovascular versus open surgical management strategies. In patients with carotid artery disease, the risk of stroke is related to plaque rupture and distal embolization. The patient underwent successful carotid stenting without periprocedural complications. Our case reports the unusual occurrence of a highly mobile atheroma as the initial presentation of carotid artery disease treated safely with percutaneous carotid artery stenting.

## Introduction

Atherosclerotic carotid artery stenosis is considered severe when stenosis is greater than 70%. The prevalence in the general population is estimated to be between 0.1 to 3% and is higher in men, Caucasians, American Indians, those with coronary artery disease, and patients older than 65 years of age [1]. There is a complex relationship between carotid artery disease, the rate of accumulation, and the risk of symptoms/stroke. The association between the degree of stenosis and the risk of stroke was previously demonstrated in the North American Symptomatic Carotid Endarterectomy Trial (NASCET). After 18 months of medical therapy without revascularization, stroke rates were 19% in those with 70% to 79% initial stenosis, 28% in those with 80% to 89% stenosis, and 33% in the 90% to 99% stenosis group [2]. Two older studies exploring the relationship between cerebral symptoms and morphological ultrasound characteristics of carotid plaque/stenosis demonstrated increased rates of clinical cerebral ischemic events with ulceration, echolucency, intraplaque hemorrhage, and high lipid content [3,4].

Diagnosing carotid artery disease typically begins with a history and physical examination. Neurologic symptoms are the predominant features associated with carotid artery disease, which include visual disturbances, dizziness, transient or permanent motor or sensory deficits, etc. The most common physical examination finding includes carotid bruits on auscultation. In the Framingham Heart Study, the calculated age-adjusted incidence of stroke in patients with cervical bruits was 2.6 times that of those without bruits [5]. 

Duplex ultrasound, CT angiography (CTA), and magnetic resonance (MR) angiography are modalities that can be used to evaluate carotid stenosis. Duplex ultrasound is the modality of choice for the initial evaluation of carotid artery disease [6,7]. Here we discuss a case regarding severe symptomatic carotid stenosis with an unusual presentation including a mobile atheroma.

## Case presentation

A 69-year-old male patient was found to have a left carotid bruit on physical exam. He had remained asymptomatic at the time of presentation. The differential diagnosis included but was not limited to carotid artery stenosis, carotid artery dissection, radiation of valvular murmurs (eg. aortic stenosis), and intracranial arteriovenous malformations. A carotid ultrasound was ordered for further evaluation, and this revealed an unusual finding of what appeared to be a mobile atheroma in the proximal left internal carotid artery. Following this result, the patient was referred to our institution for further management.

**Figure 1:**
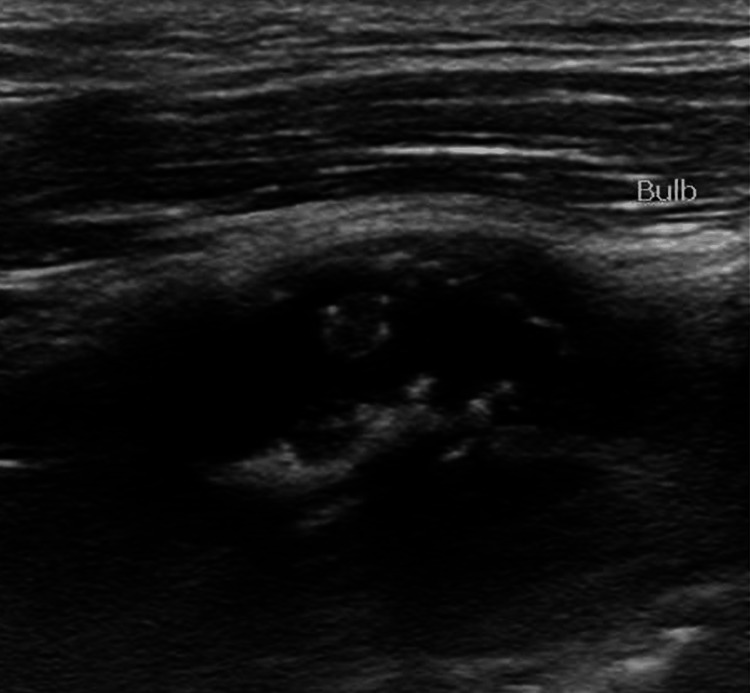
Carotid artery ultrasound (long-axis view) of the left internal carotid artery. Near the left common carotid artery bulb, there is a mobile atheroma.

**Figure 2:**
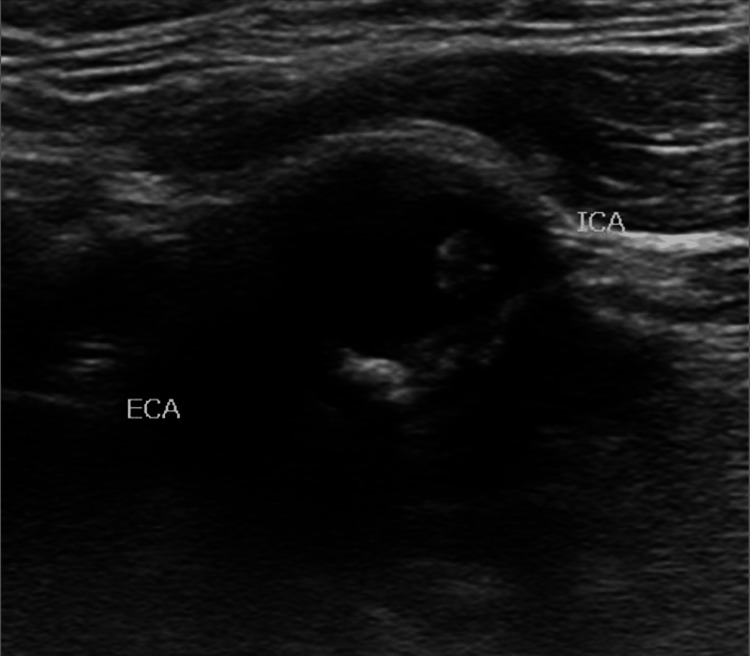
Short-axis view of the left internal carotid artery (ICA) near the left common carotid artery bulb revealing a mobile atheroma. External carotid artery (ECA) and ICA are labeled.

Short- and long-axis carotid ultrasound images of the left internal carotid artery are shown in Figures 1, 2.

**Figure 3:**
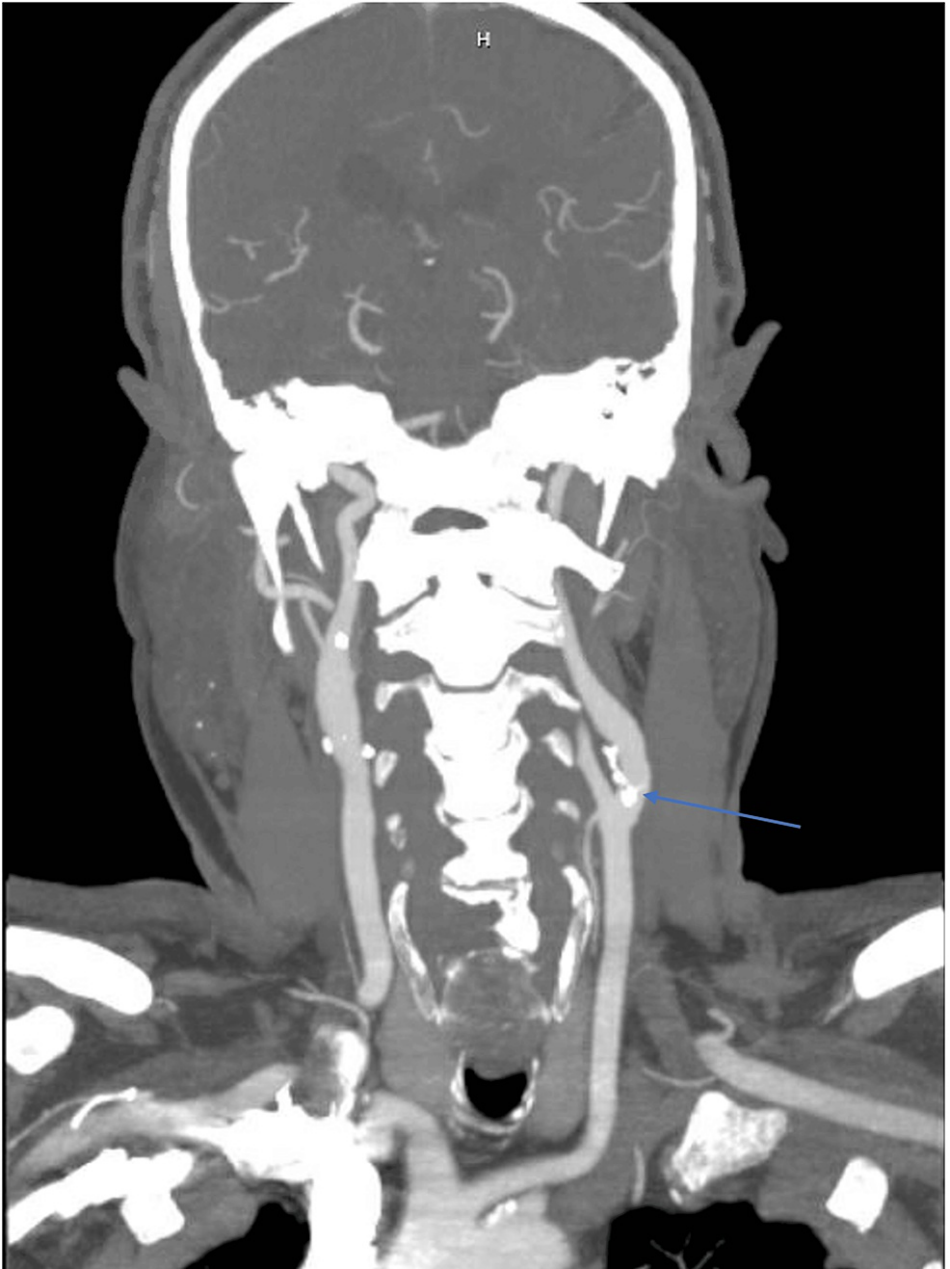
CTA of the head and neck vessels (coronal maximal intensity projection) demonstrating mixed calcified and soft plaque in the proximal left internal carotid artery protruding from the posterior wall and extending more centrally with no gross hemodynamically significant stenoses (arrow). CTA: CT angiography

**Figure 4:**
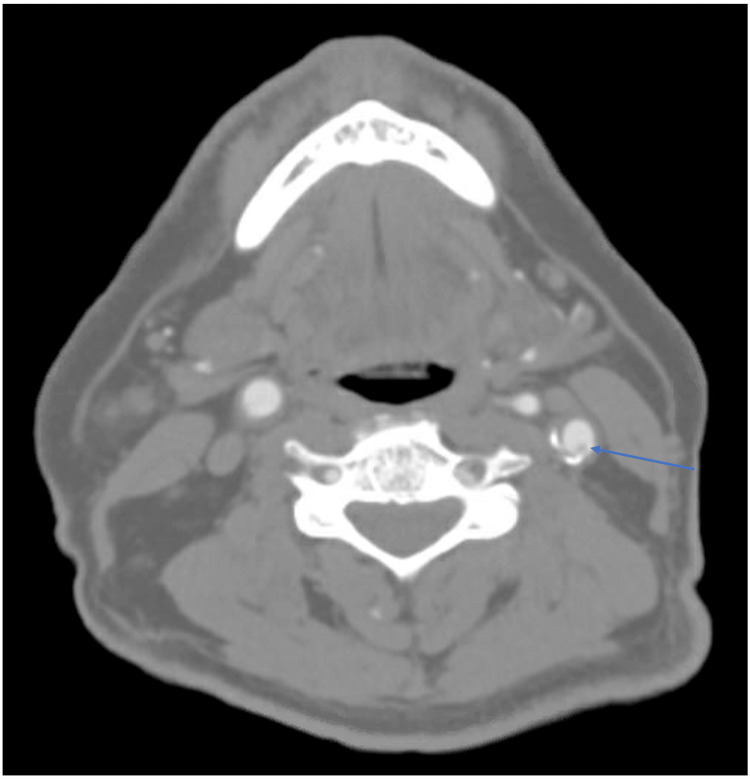
CTA of the head and neck vessels (axial view) demonstrating mixed calcified and soft plaque in the proximal left internal carotid artery protruding from the posterior wall and extending more centrally with no gross hemodynamically significant stenoses (arrow). CTA: CT angiography

A CTA of the head and neck vessels revealed mixed calcified and soft plaque in the proximal left internal carotid artery protruding from the posterior wall and extending more centrally without hemodynamically significant stenosis (Figures 3, 4).

There was no evidence of occlusion, dissection, or aneurysm otherwise.

**Figure 5:**
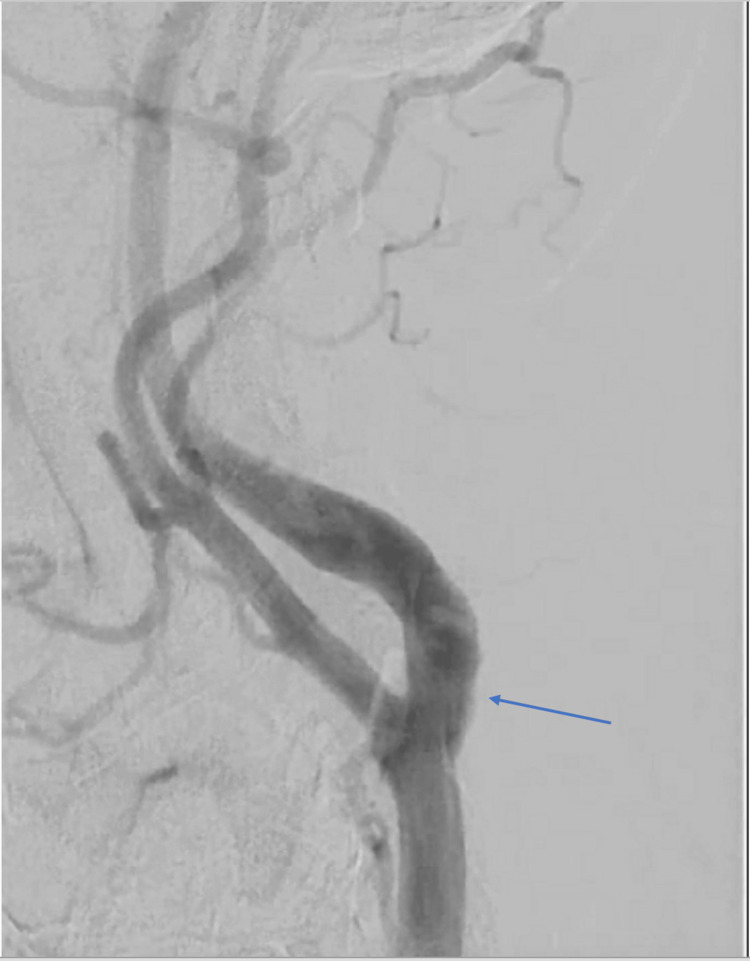
Angiographic view of the left common carotid artery bifurcating into the left internal and external carotid arteries prior to the deployment of a carotid stent in the left internal carotid artery (left anterior oblique view). The blue arrow is pointing towards the proximal left internal carotid artery.

**Figure 6:**
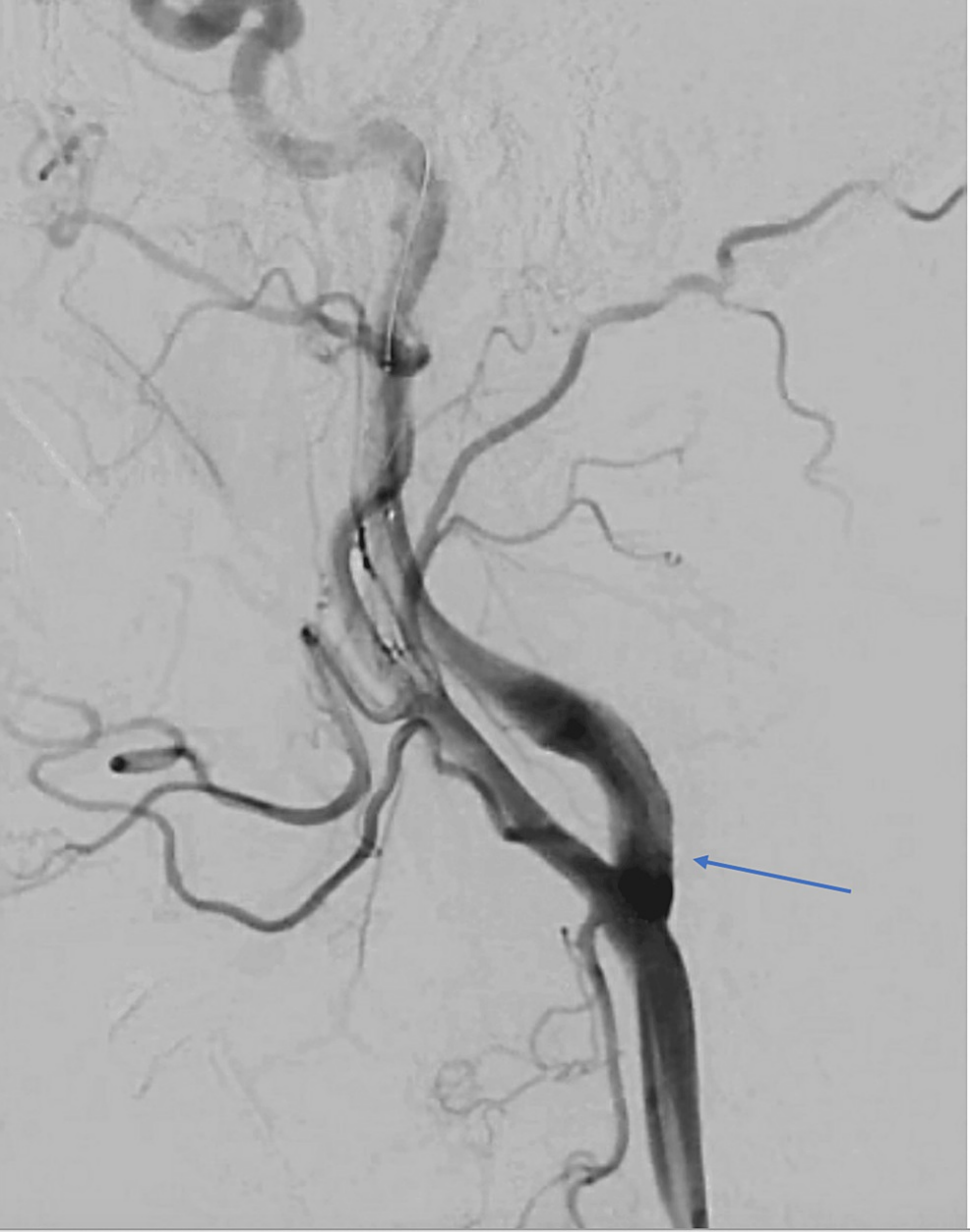
Angiographic view of the left common carotid artery bifurcating into the left internal and external carotid arteries following deployment of a carotid stent in the left internal carotid artery (left anterior oblique view). The blue arrow is pointing towards the proximal left internal carotid artery.

Discussion with other specialties in a multidisciplinary approach regarding further management was held between the patient, neurology, interventional neurosurgery, and interventional cardiology teams. The patient ultimately made the decision to proceed with a percutaneous strategy. The patient was brought to the cardiac catheterization lab. Interventional neurosurgery was present for backup in case of an acute complication relating to possible embolization. Under ultrasound guidance, the right common femoral artery was accessed. A left carotid angiography was then performed (Figures 5, 6) after a 6F Shuttle Select sheath (Cook Medical LLC, Bloomington, Indiana, United States) was placed in the left common carotid artery.

An Emboshield NAV6 embolic protection device (Abbott Laboratories, Chicago, Illinois, United States) was carefully deployed in the distal left internal carotid artery. A 7-10.0mm x 30mm x 132cm Acculink carotid stent system stent (Abbott Laboratories, Chicago, Illinois, United States) was advanced across the lesion and successfully deployed without predilation. The embolic protection device was removed. A 6F perclose vascular closure device was used for arterial hemostasis. The patient tolerated the procedure without any immediate complications. 

The patient was seen and followed up closely in the clinic a week and one month following discharge. No neurologic or cardiovascular symptoms were reported at the follow-up.

## Discussion

Mobile carotid plaques observed on carotid duplex ultrasound have previously been shown to be histologically different and associated with progressive ischemic symptoms compared to their non-mobile counterparts [8]. Despite the higher theoretical risk of embolization, data regarding the natural progression of mobile atheromas is sparse. Case reports in asymptomatic patients have suggested atheroma stabilization and patient safety with antiplatelet/anticoagulation therapy [9]. Hence, medical therapy with ultrasound surveillance and intervention being reserved for symptomatic patients is a reasonable initial approach [10]. Due to a lack of specific guidelines, a multidisciplinary shared decision-making approach was employed for our patient and the decision was made to proceed with intervention.

There have been more than 10 randomized trials comparing endarterectomy and carotid artery stenting with the largest of these to date being the Carotid Revascularization Endarterectomy versus Stenting Trial (CREST) [11]. These studies have consistently suggested that the two modalities when performed by expert operators, achieve equivalent long-term benefits. However, the procedures have differing inherent safety profiles, with carotid artery stenting patients incurring more peri-procedural minor strokes, while endarterectomy patients have more peri-procedural myocardial infarctions and develop higher rates of postprocedural cranial nerve palsy [11,12]. The decision for carotid stenting versus surgical intervention was guided by the patient's comorbidities, considering his prior history of myocardial infarction.

Distal embolic protection (DEP) poses an increased risk of embolization during the advancement of the device. Despite the elevated risk, DEP has previously been successfully employed for neuroprotection prior to stenting mobile carotid atheromas similar to our case [13]. Proximal embolic protection (PEP) in combination with DEP has been used successfully in a patient with ischemic stroke secondary to mobile carotid atheroma [14]. However, despite the reduction in ischemic lesions, the addition of PEP has not been shown to reduce periprocedural stroke, transient ischemic attack (TIA), or death [15]. Transcarotid artery revascularization (TCAR) is a novel technique that involves direct cannulation of the common carotid artery to deliver the stent while simultaneously diverting flow to the femoral vein [16]. Currently, there have been no randomized controlled trials directly comparing TCAR with other DEP modalities. Hence, the decision to proceed with DEP in our patient was guided predominantly by available technology and operator expertise. 

The type of stent used is also an important consideration in patients with mobile atheromas. Open-cell stent designs offer the advantage of increased conformity but have been associated with increased non-clinically significant embolic events compared to closed-stent designs [17]. Despite this increased risk, cases of mobile atheromas stented with open-stent design have been reported without significant clinical neurologic sequelae [13]. In our patient, as the plaque was located in a tortuous segment at the bifurcation, an open cell stent design was favored and successfully deployed.

This case report demonstrated the successful percutaneous management of a mobile atheroma that may have been considered for open surgical therapy. Future studies are needed with larger numbers of patients investigating the short- and long-term outcomes after percutaneous interventions of mobile atheromas. Additionally, comparisons of different percutaneous intervention methods comparing TCAR with other DEP modalities are warranted to aid in future decision-making.

## Conclusions

Our case reports the unusual occurrence of a highly mobile atheroma as the initial presentation of carotid artery disease, treated safely with carotid artery stenting. Mobile carotid atheromas are considered at higher risk for embolization and the decision to intervene should be guided by a multidisciplinary shared decision-making approach. Carotid stenting is a viable treatment strategy for treatment for patients with significant comorbidities, advanced age, and limited surgical options.
